# A single centre experience with sequential and concomitant chemoradiotherapy in locally advanced stage IV tonsillar cancer

**DOI:** 10.1186/1748-717X-5-121

**Published:** 2010-12-21

**Authors:** Robin J D Prestwich, Kiran Kancherla, Didem Colpan Oksuz, Deborah Williamson, Karen E Dyker, Catherine Coyle, Mehmet Sen

**Affiliations:** 1 St. James's Institute of Oncology, St. James's University Hospital, Leeds Teaching Hospitals, Leeds, West Yorkshire, UK

## Abstract

**Background:**

Chemo-radiotherapy offers an alternative to primary surgery and adjuvant therapy for the management of locally advanced stage IV squamous cell carcinomas of the tonsil.

**Methods:**

A retrospective analysis was performed of the outcomes of 41 patients with locoregionally advanced squamous cell carcinoma of the tonsil treated non-surgically at the Yorkshire Cancer Centre between January 2004 and December 2005. Due to long radiotherapy waiting times, patients received induction chemotherapy with cisplatin and 5-fluorouracil followed by either cisplatin concurrent chemoradiotherapy or radiotherapy alone.

**Results:**

Median age was 55 years (range 34-76 years) and 28 (68%) patients were male. 35/41 patients (85%) received 2 or more cycles of induction chemotherapy. Following induction chemotherapy, 32/41 patients (78%) had a clinical response. Concomitant chemotherapy was given to 30/41 (73%). All patients received the planned radiotherapy dose with no delays. There were no treatment related deaths. Six (15%) patients had gastrostomy tubes placed before treatment, and 22 (54%) required nasogastric tube placement during or after treatment for nutritional support. 17 patients required unplanned admissions during treatment for supportive care. At 4 months post treatment assessment 35 out of 41 (85%) patients achieved complete clinical and radiographic response. Median follow-up is 38 months (8-61 months). Local and regional control rate in complete responders at 3 years was 91%. Distant metastases have been found in 4 (9.8%) patients. Three year progression-free survival rate in all patients is 75%. The 3-year cause specific survival and overall survival are 75% and 66% respectively.

**Conclusion:**

Cisplatin-based induction and concurrent chemoradiotherapy provides excellent tumour control with acceptable toxicity for patients with locally advanced tonsillar cancer.

## Introduction

Head and neck squamous cell carcinomas (HNSCC) are the sixth most common cancers [1], with around two thirds of patients presenting with locally advanced disease. The treatment of advanced disease poses a major challenge in terms of balancing tumour outcomes with acceptable toxicity and maintaining organ function [2,3]. For many years primary surgery and/or radiotherapy have been the mainstay of treatment. Organ preservation using radiotherapy has been accepted as an alternative to surgery [4,5].

The role of chemotherapy has gradually emerged, and is now taking a more prominent place in treatment algorithms for locally advanced HNSCC. The use of concurrent chemotherapy has improved locoregional control, with optimal results being achieved with cisplatin [6-10]. Induction chemotherapy has been used in an attempt to gain the benefit of full therapeutic doses of chemotherapy via additive clonogen cell kill and spatial cooperation to treat distant micro metastatic disease, whilst avoiding the enhanced toxicity of concurrent treatment [11]. The potential to reduce the risk of developing distant metastases is particularly attractive as locoregional control improves with combined modality treatment. Meta-analyses have demonstrated a small survival advantage of 2% with induction chemotherapy, although cisplatin/5-FU regimens were associated with a larger benefit in the order of 5% [6]. Recently, two phase III studies have demonstrated an additional benefit with the addition of docetaxel to cisplatin/5-FU induction chemotherapy [12,13].

It has become evident that HNSCC represents a highly heterogenous group of tumours. In order to improve the therapeutic ratio of treatment it is critical to understand the varied aetiology, biology and response to treatment of tumours arising from different anatomical subsites. It is therefore essential to report the outcome of treatment for individual subsites, as opposed to simply grouping them together. In this way, it may be possible to identify tumour sites which would benefit from treatment intensification, or alternatively tumour sites with a favourable outcome for which a treatment de-escalation could be considered to limit toxicity [2,3].

The oropharynx is a common head and neck cancer subsite accounting for just over 1000 cases each year in UK [14]. Tonsillar tumours represent the most common site of origin of tumours within the oropharynx, with a steadily climbing incidence due in part to human papilloma virus [15]. Non-surgical treatment plays a major role in the management of tonsillar squamous cell carcinomas (SCC). A retrospective review [16] reported similar tumour control following primary surgery or radiotherapy in tonsillar cancer; however, the risk of severe or fatal complications is higher for patients treated surgically (> 20%) than those treated with RT (2% - 11%). Currently, the choice of primary surgical or non-surgical treatment depends upon local expertise, physician and patient preference.

Long radiotherapy waiting times have been a major issue in UK [17]. In our regional cancer centre, radiotherapy waiting times of around 3 months were prevalent at the time of this series, in common with many other UK centres [18]. Delays in commencing radiotherapy have associated with a decrease in local control rates [19]. Locally advanced HNSCC were routinely treated with induction cisplatin/5-FU chemotherapy in order to avoid stage progression of tumours whilst awaiting treatment. Concurrent chemotherapy was additionally administered depending upon tumour factors, patient fitness and comorbidity.

Here we present the outcomes for patients with locally advanced stage IV SCC of the tonsil managed with induction chemotherapy followed by radical (chemo-)radiotherapy. These data, in patients treated in routine clinical practice, demonstrate the feasibility of adding induction chemotherapy without compromising subsequent (chemo-)radiotherapy, and obtaining high rates of tumour control without the need for surgery.

## Materials and methods

From 1st January 2004 to 31st December 2005 patients with a diagnosis of locally advanced stage IV tonsil squamous cell carcinoma without distant metastases who were treated at the Yorkshire Cancer Centre were identified from the radiotherapy database. Patients who received radical surgery and post-operative radiotherapy were excluded from analysis. Data was obtained by a retrospective review of the clinical notes, radiotherapy and chemotherapy records, and the oncology patient database. All patients were treated under the auspices of the specialist Head and Neck multidisciplinary team, following a written protocol. Within this protocol, all patients were investigated and staged with nasoendoscopy, biopsy, computed tomographic (CT) scanning and/or magnetic resonance imaging (MRI) of head and neck region, CT of thorax. Physical examination, dental, dietary, speech and language assessment, full blood count, electrolytes, liver and kidney function tests were routinely performed before initiation of treatment. The disease was staged according to the 2002 classification of the American Joint Committee on Cancer Staging. All patients were treated with induction chemotherapy followed by concurrent chemoradiotherapy or radiotherapy. Outcomes in terms of toxicity, site of relapse, disease free survival (DFS), and overall survival were determined by a retrospective notes review, analysis of radiotherapy treatment records, and oncology databases. Toxicity was routinely documented prospectively using the NCIC-version 3.0 grading system for chemotherapy toxicity, and the RTOG system for radiotherapy toxicity. Waiting time for radiotherapy was defined as the number of days from the clinic at which a decision was made to treat with radiotherapy to the first day of radiotherapy.

### Induction chemotherapy

Standard induction chemotherapy consisted of 1-4 cycles of cisplatin 80 mg/m^2 ^day 1 and 5-fluorouracil (5 FU) 800 mg/m^2 ^days 2-5, three weekly. Patients underwent clinical, haematological and biochemical assessment prior to each cycle; toxicity was prospectively recorded. Further cycles were only given after satisfactory toxicity assessment by medical staff. The number of cycles administered depended upon the wait until commencement of radiotherapy, tumour response and toxicity.

### Radiotherapy

All patients were treated with 3-dimensional conformal radiotherapy. Patients were simulated supine using an individualized neck support and Perspex shell for immobilization. CT images for treatment planning were obtained at 2-5 3 mm intervals from the skull vertex to below the carina. The CT data were loaded into the Helax-TMS VG-1B treatment planning system. The target volume included primary site and bilateral level Ib, II, III, IV, V lymph nodes and retropharyngeal lymph nodes. Treatment was planned with a two phase technique of two parallel opposed photon fields, with a matched anterior neck field. The posterior border of the lateral 6MV photon fields was brought anterior to spinal cord to avoid cord toxicity (after 39.75 Gy in 13 fractions in the hypofractionated regimen or 44 Gy in 22 fractions in the conventionally fractionated regimen), and matched electron fields were applied to the posterior neck. Due to prevalent waiting times, radiotherapy was booked prior to commencement of chemotherapy and schedules based upon clinicians' judgement/preferences and not upon chemotherapy responses. Two general schedules were routinely used at the time: i) a conventionally fractionated regimens of 65-70 Gy in 30-35 fractions over six and a half to seven weeks with 50 Gy in 25 fractions over five weeks to the matched anterior neck, and ii) an accelerated hypofractionated regimen of 55 Gy in 20 fractions over four weeks with 40 Gy in 15 fractions over three weeks to the matched anterior neck. During radiotherapy, patients were reviewed twice weekly, by a multidisciplinary team involving clinician, nurse, dietician and speech and language therapy team.

### Concomitant chemotherapy

Cisplatin 80 mg/m2 days 1 and on the final day of radiotherapy was used for accelerated hypofractionated radiotherapy regimen. Cisplatin 100 mg/m2 days 1, 22 and 43 was used for the conventionally fractionated regimen. Cisplatin was delivered with 2 litres pre-hydration and 2 litres post-hydration with normal saline during an overnight inpatient stay. Carboplatin (area under curve 4) was substituted for cisplatin if creatinine clearance was < 55 ml/min calculated by the Cockroft and Gault formula and confirmed if time permitting by isotopic GFR assessment. Full blood count, urea, serum creatinine were checked prior to each course of chemotherapy.

### Response assessment and Follow-up

After completion of therapy, each patient was followed up clinically after 4-6 weeks to assess acute toxicity. Tumour response was assessed 4 months after the completion of the treatment. Evaluation of tumour response was routinely evaluated where indicated by a detailed clinical examination of the head and neck, nasoendoscopy and CT or MRI imaging of the primary site and the neck. An examination under anaesthetic and biopsies were performed in the event of clinical, nasoendoscopic or radiological abnormalities. Patients with less than a complete response were evaluated for surgery. Patients who were considered suitable for surgery by the multi-disciplinary team underwent salvage surgery of primary site and/or neck dissection. Subsequently, patients were followed up with physical examination, and flexible endoscopy every 6-8 weeks in the first year after treatment, every 3 months for an additional 2 years, and every 6 monthly until discharge at 5 years.

### Statistical analysis

The following endpoints were used for assessment: induction chemotherapy response, overall treatment response, progression-free survival (PFS), locoregional recurrence-free survival (LRFS), distant metastasis-free survival (DMFS), overall survival (OS) and cause specific survival (CSS). PFS, LRFS, DMFS, OS and CSS were analyzed using Kaplan-Meier product limit curves. Time was measured from the date of diagnosis. Patients who relapsed but for whom salvage therapy was successful were still considered to have experienced failure at the time of event occurrence. In the overall survival estimates, deaths due to all causes are included in the calculations. Significance of differences between survival curves was calculated by the log rank test. A p value of 0.05 or less was declared statistically significant. Univariable analysis was performed stratified by tumour stage (T stage), nodal stage (N stage) and treatment (induction chemotherapy followed by concurrent chemoradiotherapy or induction chemotherapy followed by radiotherapy alone).

## Results

45 patients were identified who were treated with radiotherapy for locally advanced stage IV tonsillar squamous cell carcinoma. Four (9%) of these 45 patients were treated with primary surgery and received post-operative radiotherapy and were excluded from analysis. Median age of the remaining 41 patients was 55 years (range 34-76 years) and 28 (68%) patients were male. All 41 patients had pathologically confirmed squamous cell carcinoma; 1 (2%) was grade 1, 12 (29%) were grade 2, and 28 (68%) were grade 3. Patient characteristics are shown in Table 1. All patients were non-metastatic stage IV. Twenty-five (61%) patients had T3-4 primary disease, while 39 (95%) had N2-3 lymph node disease. Respective T and N stage distributions are detailed in Table 2.

**Table 1:** Patient characteristics

	N	%
**Gender**		

Female	13	31.7

Male	28	68.3

**Age (yrs)**		

≤60 yrs	31	75.6

>60 yrs	10	24.4

**Table 2:** Tumour characteristics

	N classification				
	
T classification	N0	N1	N2	N3	Total
**T1**	-	-	3	2	5

**T2**	-	-	8	3	11

**T3**	-	-	3	3	6

**T4**	1	1	15	2	19

**Total**	1	1	29	10	41

The median time between first clinic consultation to the start of radiotherapy was 77 days (range 50-122 days). All patients received cisplatin/5 FU induction chemotherapy during this delay. 6 (14%) patients received one chemotherapy cycle, 23 (56%) received two cycles, 10 (24%) received three cycles and 2 (6%) patients received four cycles. Fourteen (34%) of patients required an alteration or dose reduction of chemotherapy treatment. Following induction chemotherapy clinical response assessment indicated 32/41 patients (78%) had either a complete or partial response. The responses to induction chemotherapy are summarized in Table 3. Several different radiotherapy schedules were used. 9 (22%) of patients received an accelerated hypofractionated schedule of 55 Gy in 20 fractions over 4 weeks. The remaining 32 patients received conventionally fractionated regimens (10 patients received 70 Gy in 35 fractions, 10 received 68 Gy in 34 fractions, 8 received 66 Gy in 33 fractions and 4 patients received 65 Gy in 30 fractions. Due to radiotherapy waiting times, radiotherapy schedules were booked prior to the commencement of chemotherapy and were hence based upon clincians' judgement/preference rather than response to induction chemotherpay. The median time from the adminstration of the final cycle of induction chemotherapy to the first fraction of radiotherapy was 21 days, with a range of 10-42 days.

**Table 3:** Tumour responses assessed clinically after induction chemotherapy, and clinically and radiologically 4 months after completion of radiotherapy

	Complete response N (%)	Partial response N (%)	Stable disease N (%)
**After induction chemotherapy**	4 (10%)	28 (68%)	9 (22%)

**4^th ^month after the radiotherapy**	35 (85%)	6 (15%)	-

Chemotherapy was administered concomitantly with radiotherapy to 30 of 41 patients (73%). The decision whether to administer concomitant chemotherapy was made by the treating Clinical Oncologist, based upon tumour and patient factors. These included age, performance status, response and toxicity with induction chemotherapy. The 11 patients who did not receive concomitant chemotherapy had a median age of 58 (range 48-76); 8 of 11 had T3/4 disease (T4 n = 6) and 10 of 11 had N2/3 disease (N3 = 1). The 30 patients treated with concomitant chemotherapy had a median age of 54 (range 43-74); 17 of 30 had T3/4 disease (T4 n = 13) and 29/30 had N2/3 disease (N3 = 9). 4 of the 9 patients receiving hypofractionated radiotherapy with 55 Gy in 20 fractions over 4 weeks received concomitant chemotherapy. 26 of 32 patients receiving conventionally fractionated radiotherapy received concomitant chemotherapy.

Of the 30 patients treated with concomitant chemotherapy, 19 received only one of the planned cycles of concurrent chemotherapy, while 11 of the 30 patients completed two cycles of concurrent chemotherapy and no patient received three. All of the 4 patients treated with 55 Gy in 20 fractions over 4 weeks received only one cycle of concomitant chemotherapy. Of the 26 patients receiving concomitant chemotherapy with conventionally fractionated radiotherapy, 15 (58%) received one cycle of chemotherapy and 11 received 2 cycles (42%). Radiation therapy was completed in all patients without any delays greater than 3 days. There were no treatment related deaths.

### Treatment Response

At 4 months post treatment assessment 35 (85%) patients achieved complete clinical and radiographic response (Table 3). The six (14%) remaining patients achieved a partial response and were evaluated for salvage surgery. Among these patients with a partial response, neck dissections were performed in 2. Both patients had had stable disease after induction chemotherapy and neck dissection pathology showed extensive nodal involvement with extra capsular spread. Both patients died with locoregional recurrence and one of them developed lung metastasis. The remaining 4 patients died with locoregional progression, with a median survival of 10 months (range 8-14).

### Survival outcomes

Median follow-up of all patients is 38 months (range 8-61 months). 27 (66%) patients remain alive, with a median follow-up of 43 months (range 36-61 months). Four patients (11%) have died during follow up following a complete response to treatment without any evidence of subsequent disease recurrence. One of these patients died following a carotid blow out without evidence of disease recurrence on post-mortem; the other three deaths were due to myocardial infaction, Alzheimer's disease and a second primary tumour (adrenal).

**Figure 1:**
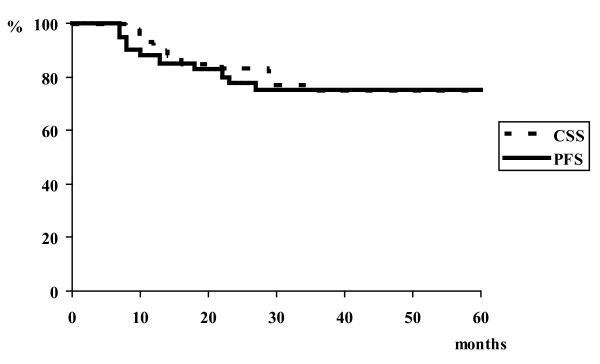
**Cause specific survival and progression-free survival in stage IV tonsil**.

Local and regional control rate in complete responders at 3 years was 91% and median time to local and/or regional recurrence was 20 months (range 13-23 months). Of the 35 patients with complete remission at four month post-treatment assessment, one experienced an isolated local failure, one an isolated regional failure, one local and regional failure, one locoregional failure with distant metastases. Among the three patients with isolated local and/or regional recurrence, one has undergone salvage surgical resection after 13 months disease free interval. Distant metastases were detected in 4 (10%) patients with a median 13 months of follow up (range 7-27 months). Three of these four patients did not experience locoregional failure. Three year distant metastases free survival rate was 89%. Lung was the distant metastases site in all patients. Three years progression-free survival rate in all patients is 75%. The 2 and 3-year overall survival rate is 76% and 66% respectively, and the 2 and 3-year cause specific survival rates are 80% and 75% respectively. Overall survival outcomes are lower than cause specific outcomes due to the 4 deaths during follow up without evidence of active disease. Figure 1 shows the progression-free and cause specific survival rates.

### Prognostic factors

Univariable analysis revealed that the 3-year progression-free survival and cause specific survival were significantly better for patients with T1 and T2 disease compared to T3 and T4 disease, respectively (p = 0.004 and p = 0.004). However, nodal stage and treatment type did not show a significant association with progression-free survival, distant metastasis-free and cause specific survival. The association of T stage, nodal stage and treatment type with PFS, DMFS, and CSS are given in Tables 4.

**Table 4:** Univariate analysis for progression-free survival (PFS), distant metastasis-free (DMFS) survival and cause specific survival (CSS) rates

		PFS		DMFS		CSS	
	**N**	**3 years %**	**P**	**3 years %**	**p**	**3 years %**	P

**T stage**							

T1+T2	16	100		100		100	
			**0.004**		0.07		0.004
					
T3+T4	25	59		81		58	

**Nodal stage**							

N0-1	2	100		100		100	
			0.45		0.65		0.46
					
N2-3	39	74		89		73	

**Treatment type**							

Induc CT-CTRT	30	72		89		72	
			0.6				0.59
			
Induct CT-RT	11	82		91	0.93	82	

### Acute Toxicity

#### Induction chemotherapy

Grade 3 neutropenia occurred in 4 patients, 2 experienced grade 3 mucositis.

#### (Chemo-)radiotherapy

Among the 30 patients who had concomitant chemoradiotherapy, there was one case each of grade 3 vomiting and of febrile neutropenia requiring admission. In 6 of 30 patients, carboplatin was substituted for cisplatin owing to renal impairment. At the end of radiotherapy, in the whole cohort of 41 patients RTOG grade 3 skin reaction was documented in 31, and RTOG grade 3 mucosal toxicity in 29 patients. In the 9 patients receiving 55 Gy in 20 fractions over 4 weeks, 6 experienced RTOG grade 3 skin toxicity and 7 experienced RTOG grade 3 mucositis. In the remaining 32 patients receiving conventionally fractionated radiotherapy, 25 experienced RTOG grade 3 skin toxicity and 22 had RTOG grade 3 mucositis.

Six (15%) patients had gastrostomy tubes placed prophylatically before treatment. 22 (54%) of patients required nasogastric tube (NG-tube) placement during (n = 17), or after (n = 5) treatment for nutritional support. More than 10% weight loss during therapy was seen in 10 (24%) patients. Seventeen patients required admission for supportive care or nutrition during the radiotherapy and 14 of these were treated with concomitant chemoradiotherapy. 4 out of 9 (44%) patients receiving 55 Gy in 20 fractions over 4 weeks and 19 out of 32 (59%) patients receiving conventionally fractionated radiotherapy required admission.

#### Late Toxicity

Among 27 surviving patients, as a long-term treatment-related complication 2 patients have been recorded as having grade 3 dysphagia. At present no patient is gastrostomy tube dependent. Trismus has been documented in 4 patients. Four patients developed soft tissue or osteoradionecrosis. One of them received 3 courses of induction chemotherapy followed by concomitant chemoradiotherapy died due to soft tissue, carotid artery necrosis 7 months after the therapy.

## Discussion

Concurrent chemo-radiotherapy has been widely adopted as the standard of care for locally advanced HNSCC [6,7]. Cisplatin is the chemotherapy agent of choice, with studies showing a 5-12% improvement in long term survival with standard or altered fractionation regimens [6,8]. The improvement in survival comes at the expense of increased acute and late toxicity [8,20].

Induction chemotherapy followed by sequential radiotherapy is an alternative approach to concurrent treatment which has been shown to have a survival benefit in locally advanced HNSCC [21-23]. Although induction chemotherapy has only a minimal survival benefit of 2% in a large meta-analysis, the combination of cisplatin and 5-FU was associated with a 5% survival benefit [6]. Two phase III studies have subsequently demonstrated that induction chemotherapy with docetaxel, cisplatin and 5-FU (TPF) offers a significant survival advantage over induction with cisplatin and 5-FU [12,13]. In patients with unresectable HNSCC, induction with TPF resulted in a 27% reduction in the risk of death after a median of 32 months follow-up [13]. Similarly, in the study based in the US, 3 year overall survival with TPF induction was 62% compared with 48% in the cisplatin and 5-FU induction group [12].

One major concern with the addition of induction chemotherapy is that it may compromise the ability to deliver radiotherapy. In the EORTC/TAX323 study examining induction chemotherapy, it is notable that only 120 of 179 patient receiving cisplatin and 5-FU, and 129 of 173 patients receiving TPF, ever received radiotherapy [13]. The failure of such a significant proportion of patients to ever receive the potentially curative part of the treatment schedule is a major concern with induction chemotherapy. A further potential disadvantage of induction chemotherapy is that the ability to deliver concurrent chemotherapy may be compromised.

The role of systemic treatment in addition to radiotherapy in locally advanced HNSCC continues to develop. Concurrent chemo-radiotherapy remains a standard of care, while induction chemotherapy has clear evidence of efficacy. However, it remains uncertain whether combining induction with concurrent chemotherapy takes advantage of the benefits of both treatments. Studies are currently underway to investigate the potential superiority of induction chemotherapy followed by concurrent chemoradiotherapy compared with concurrent chemoradiotherapy alone.

Radiotherapy waiting times have been a major issue in the UK [17,18], particularly for HNSCC with rapid tumour doubling times. During the 2004-5 period reported here, protracted radiotherapy waiting times of 3 months were common. Therefore, induction chemotherapy was routinely offered to our patients. This era was prior to the publication of the data demonstrating the superiority of induction with TPF [12,13], and cisplatin and 5-FU was the standard induction regimen. The patients with tonsil carcinoma reported here received between 1 and 4 cycles prior to radiotherapy, although the total number depended upon the wait for radiotherapy to commence, along with tolerance and response to treatment. The radical radiotherapy schedules in use at the time were either a conventionally fractionated 65-70 Gy in 30-35 fractions, or a hypofractionated accelerated regimen of 55 Gy in 20 fractions. The latter regimen reflected historical radiotherapy practice within the UK, and also a pragmatic response to waiting times. Following guidance from the Royal College of Radiologists, the hypofractionated schedule is no longer employed in our centre for locally advanced HNSCC [24].

With implementation of various measures our radiotherapy waiting times have now fallen to 4 weeks in line with the national radiotherapy waiting times target. Nevertheless, in addition to providing data on the use of induction chemotherapy to compensate for protracted waiting times for radiotherapy, this series provides important data on the tolerability and efficacy of induction chemotherapy followed by radiotherapy ± concurrent chemotherapy outside the setting of clinical trials. Subjects within clinical trials are almost inevitably a fitter selected subset of patients. A major issue with the chemo-radiotherapy trials is whether the results, based upon selected fit patients, can be successfully applied to patients encountered in routine clinical practice. The results of institutional series of patients treated outside clinical trials are invaluable in exploring these issues.

The series of 41 patients reported here, treated in 2004-5, demonstrates that induction chemotherapy can be successfully combined with concurrent chemoradiotherapy, without excessive toxicity. Radiotherapy commenced promptly at a median of 21 days (range 10-42) following the adminstration of the final cycle of radiotherapy. Therefore, induction chemotherapy did not preclude the prompt delivery of radiotherapy. Notably, by contrast with the EORTC/TAX323 trial [13], all patients in this series completed radiotherapy as planned. It should be noted that the dose of induction chemotherapy (cisplatin 80 mg/m2 and 5-FU 800 mg/m2 days 2-5) is lower than that used in the control arm of the EORTC/TAX323 study (cisplatin 100 mg/m2 and 5-FU 1000 mg/m2 days 1-5) [13]. In addition 70% of patients in our series received only 1-2 cycle of induction chemotherapy compared with the 3-4 cycles commonly delivered within trial protocols [6,12,13]. The lower number of cycles delivered were due the pragmatic utilisation of induction chemotherapy due to radiotherapy waiting times. Although this may now be regarded as suboptimal induction chemotherapy, the reduced dose and lower number of cycles delivered may have particular importance in successfully delivering subsequent radiotherapy. Gaps in the delivery of radiotherapy for HNSCC are known to be detrimental to outcome [25]. No patient in this series experienced a gap of 3 days; this compares with approximately one fifth of patients experiencing gaps in the delivery of radiotherapy in concomitant chemoradiotherapy trials [26,27].

Concomitant chemotherapy was given to nearly three-quarters of the patients in our series. The predominant reason for not giving concomitant chemotherapy to the remaining patients was limited performance status due to progressive symptoms in non-responders to induction chemotherapy; therefore it can be concluded that induction chemotherapy did not compromise patient fitness to commence definitive concurrent chemoradiation. Two-thirds of patients were able to receive only one cycle of concomitant chemotherapy due to toxicity. Compliance is a common problem noted with standard concurrent cisplatin regimens, with nearly one third of patients not receiving all concurrent chemotherapy cycles [28]. Several centres have now adopted two cycles as standard concomitant treatment due to poor compliance and toxicity [26,29]. In our series, no patient who was treated with hypofractionated radiotherapy 55 Gy in 20 fractions over 4 weeks received more than one cycle of concurrent chemotherapy. In our experience it is uncommon to be able to deliver more than once cycle of concurrent chemotherapy with hypofractionated radiotherapy due to significant acute toxicity of the radiotherapy schedule. However, only 11 of the 26 (42%) patients receiving conventionally fractionated concomitant chemoradiotherapy received 2 cycles. Decisions on whether to administer further cycles of concomitant chemotherapy are based upon clinical assessment of the patients; potential reasons for not administering further concomitant chemotherapy include deteriorating patient fitness, severity of radiotherapy toxicity including mucositis, and previously severe chemotherapy toxicity. In our practice we would aim to deliver further chemotherapy if there was a reasonable expectation that this would not lead to gaps in the delivery of radiotherapy. It is unclear whether the failure to achieve 2 cycles of concurrent chemotherapy in the majority of patients was due to the overall toxicity of the concurrent approach or due to cumulative toxicity from induction chemotherapy. The number of cycles of concurrent chemotherapy delivered may be considered inferior to that achieved in clinical trials. However, this may reflect differences in patients treated within and outside of clinical trials. For example, clinical trials commonly exclude patients over 70 whilst the series presented here includes patients receiving concomitant chemoradiotherapy up to the age of 74.

The overall toxicity of induction chemotherapy followed by (chemo)-radiotherapy appears acceptable. There were no on-treatment deaths; the patient who died 7 months after treatment with a carotid blow out without evidence of disease was the only death which may have been treatment-related. As would be expected, the majority of patients required enteral feeding during or shortly after completing treatment. However, on follow-up only 2 of 27 surviving patients had grade 3 dysphagia and none was gastrostomy-dependant. These data compare favourably with other chemoradiotherapy series; for example in a pooled analysis of three RTOG trials long term feeding tube dependence was 13% [30].

The tumour outcome of the patients presented here is excellent, with 85% of patients achieving a complete tumour response 4 months after completion of therapy. The timing of post-treatment response assessment varies between centres. The 4 month timepoint used here is intended to allow adequate time for post-radiotherapy response to be complete. In line with this concept, a recent study has shown that an 8 week response assessment is too early, with more complete responses being seen at 8 months than 8 weeks post-treatment [29]. For our cohort of 41 patients, 3 year cause-specific survival was 75%, and 3 year overall survival of 66%. Importantly in this context, in locally advanced HNSCC 3 year overall survival has been shown to be a good surrogate for 5 year survival [31].

The 3 year PFS for patients receiving induction chemotherapy followed by radiotherapy alone was 82% compared with 72% for those treated with induction chemotherapy followed by concomitant chemoradiotherapy (Table 4). This difference is not statistically significant (p = 0.6). The expectation would be for a superior PFS outcome for patients receiving concomitant chemotherapy. However, due to the small numbers of patients in the group without concomitant treatment (n = 11), it is not appropriate to draw conclusions regarding the benefit of concomitant chemotherapy based upon this subgroup comparison.

Table 5 presents the results of this and other sequential chemoradiotherapy studies. Our induction regime is almost identical to that used by Royal Marsden Hospital [32]. Both the studies used similar doses and number of cycles resulting in overall response in over three-quarters of the patients. Toxicity was acceptable and there were no treatment related deaths. Overall survival (OS) in our study was 66% at 3 years. This figure is superior to that reported at 2 years by some studies using sequential therapy [12,32,33], and similar to that in other series [34-36]. Whilst it is tempting to compare our results with other published series, differences in locoregional control and overall survival are likely to be heavily influenced by the patient population and tumour stage and tumour subsites included.

**Table 5:** Summary of induction chemotherapy followed by (chemo)-radiotherapy

	Leeds	RMH, UK (19)	Posner et al (15)	Hitt et al (16)	Vokes et al (20)	Machtay et al (21)	Urba et al (22)
Sequential theapy (IC + CRT)	**IC**: PF 1-4 cycles **CRT **(70 Gy in 35# Cisplatin100 m g/m2 day 1, 22, 43)/55 gy in20# Cisplatin day 1, 28	**IC:**P(75 mg/m 2)5 Fu(1000 mg/m2 for 4 days)-2 cycles + **CRT **:65 Gy in 30# with cisplatin 100 mg/m2 on day 1 & 29)	Control arm: **IC: **cisplatin (100 mg/m2) 5 FU(1000 mg/m2/day)-5 days **CRT: **70-74 Gy with weekly carboplatin AUC 1.5	Control arm: **IC**:3 cisplatin100 m g/m2 5-FU1000 mg/m2-5 days-3 cycles **CRT**: 70 gy IN 35# Cisplatin100 mg/m2 on day 1, 22, 43	**IC: **Paclitaxel/carbo platin weekly × 6 followed by **CRT: **paclitaxel, 5-FU, hydroxyurea and twice daily radiation therapy every other week	**IC: **caboplatin/pa clitaxel-2 cycles **CRT**: 70 in 35f with Concurrent Weekly paclitaxel Adjuvant chemo(2 cycles of carbo/taxol) + neck dissection in N2/N3 patients	**IC: **Cisplatin 100 mg/m2, 5 FU 1000 mg/m2 5 days-2 cycles **CRT: **72 Gy + cisplatin 100 mg/m2 day 1, 22, 43

Response	IC: 78%(overall)	IC: 76%(overall)	IC: 64% (overall)	IC: 68%(overall)	IC: 87%(overall)	IC:89%(over all)	IC: 76%(overall)
	CRT: 85%(CR)	CRT: 79%(CR)		CRT: 78% (CR)	CRT:82% (CR)	CRT:90% (CR)	CRT:54% histological CR

Overall survival (OS), disease free survival (DFS)	65%(3 YR OS)	63% (2 YR OS)	48% (3 YR OS)	61.5% (2 YR OS)	70% (3 YR OS)	70% (3 YR OS)	64%(3 YR OS)
	75%(3 YR DFS)	68% (2 YR DFS)			80% (3 YR DFS)		

Logo-regionalcontrol (LRC)	91% in complete responders at 3 yrs	71% at 2 yrs	62%	NR	94% (2 YR LRC)	82% at 3 YRS	NR

Metastasi s-free survival	89% AT 3 yrs	91% at 2 yrs	91%	NR	93% AT 2 YRS	81% ay 3 YRS	NR

Toxicity-Acute (AC), Late (LT) Gr3/4 only	**IC: **neutropenia 10%, mucositis 5% **CRT: **75% skin, 70%mucositis, dysphagia 63% **Late**: 24%	**IC**: neutropenia 5%, n&v 3% **CRT: **mucositis 60%, dysphagia 72%; **Late**: 8%	**IC**: neutropenia 56%, mucositis 27% **CRT: **mucositis38%, dysphagia 24%	**IC**: neutropenia 36%, mucositis(gr2-4) 53% **CRT**: 4 toxic deaths	**IC**:36%neutropenia **CRT**: 76% mucositis, 61% skin	**CRT: **98%mucositis **Late**: 24% Treatment mortality: 4%	**IC**: 29% grade4 **CRT**:19% grade4 Haematological

No of patients	41	145	246	193	69	53	59

Cancer site/staging	All Tonsil All stage 4	Oropharynx 54% Stage4 60%	Oropharynx 53% Stage 4 81%	Oropharynx 35% Stage 4 83%	Oropharynx 44% Stage 4 96%	All Oropharynx Stage 4 65%	Oropharynx 62%tongue base Stage 4 58%

HPV-16 is recognised as a major aetiological factor in the development of oropharyngeal carcinomas [15], although the proportion due to HPV varies widely between geographical areas [37]. The presence of HPV-16 is a powerful favourable prognostic factor for both disease control and overall survival [37-39]. In a randomised trial comparing accelerated versus conventional concomitant chemoradiotherapy in patients with stage III/IV oropharyngeal squamous cell carcinoma, 3 year overall survival was similar in both arms (70 v 64%, non-significant difference). However, 3 year overall survival for HPV positive tumours was 82% versus 57% for HPV negative tumours [39]. It remains to be determined whether HPV is a predictive marker allowing selection of particular therapeutic strategies [37]. The absence of data regarding the prevalence of human papilloma virus (HPV) within our cohort of patients with squamous cell carcinoma of the tonsil represents a limitation of our study. As with other studies [32-36], this limits the comparison of outcomes between series. The optimal methodology for the detection of HPV within tumour material is controversial, with assays including in situ hybridisation, polymerase chain reaction (PCR) and immunohistochemistry for p16 as a surrogate marker [37]. These discussions are currently under investigation in our institution.

The role of routine neck dissection after chemoradiotherapy continues to be debated. Some reports [40,41] have found no survival advantage with neck dissection in patients who achieved complete response following chemoradiotherapy. In addition, there is a higher subjective morbidity in patients undergoing neck dissection [40]. None of the patients in our series with a complete response following chemoradiotherapy underwent neck dissection; only one of these 35 patients subsequently developed an isolated nodal recurrence and subsequently succumbed to his disease. These data support the view that a neck dissection can be safely avoided in the absence of macroscopic residual disease. Further clarification of this issue will be provided by the UK National Cancer Research Institute PET neck study which is currently recruiting to investigate whether neck dissection can be safely avoided in locally advanced HNSCC with N2 or N3 nodal disease who achieve complete locoregional response following chemoradiotherapy.

The choice of treatment modality for the management of locally advanced tonsillar cancer remains controversial and varies between centres, some preferring primary surgery and others non-surgical treatment [16]. The good outcomes in terms of disease control and acceptable toxicity presented in this series provide support for a non-surgical approach to treatment.

In summary, the non-surgical treatment of tonsillar squamous cell carcinomas offers very high rates of locoregional control and overall survival. Induction cisplatin-based chemotherapy can be combined with radical (chemo-) radiotherapy, without a detrimental effect upon radiotherapy delivery, and acceptable toxicity. Further issues remain to be addressed, including the necessity of both induction and concurrent treatment for tonsillar tumours with an overall favourable outcome; reduced treatment intensity may be possible to reduce toxicity without compromising tumour control. The future of improving the outcomes of head and neck therapy, in terms of both tumour control and toxicity, may lie in our ability to individualise treatment. This will involve the identification of predictive and prognostic markers, including HPV status, and understanding the biological behaviour and outcome of individual tumour subsites.

## Competing interests

The authors declare that they have no competing interests.

## Authors' contributions

RJDP: Data analysis, interpretation, manuscript preparation and approval; KK: Data analysis, interpretation, manuscript preparation and approval; DCO: Data analysis, interpretation, manuscript approval; DW: Data collection, analysis, manuscript approval; KED: Original Concept, Manuscript approval; CC: Original Concept, Manuscript approval; MS: Original concept, data interpretation, manuscript approval.

All authors read and approved the final manuscript.
